# Supplementary material for: Identification of Key Genes and Regulatory Pathways in Multiple Sclerosis Brain Samples: A Meta-Analysis of Micro-Array Datasets
Source: Int J Mol Sci. 2023 May 27;24(11):9361. doi: 10.3390/ijms24119361 (PMC10253947; doi:10.3390/ijms24119361)
Supplement: Supplementary file 1 [file ijms-24-09361-s001.zip › ijms-2351706-supplementary figures.pdf]

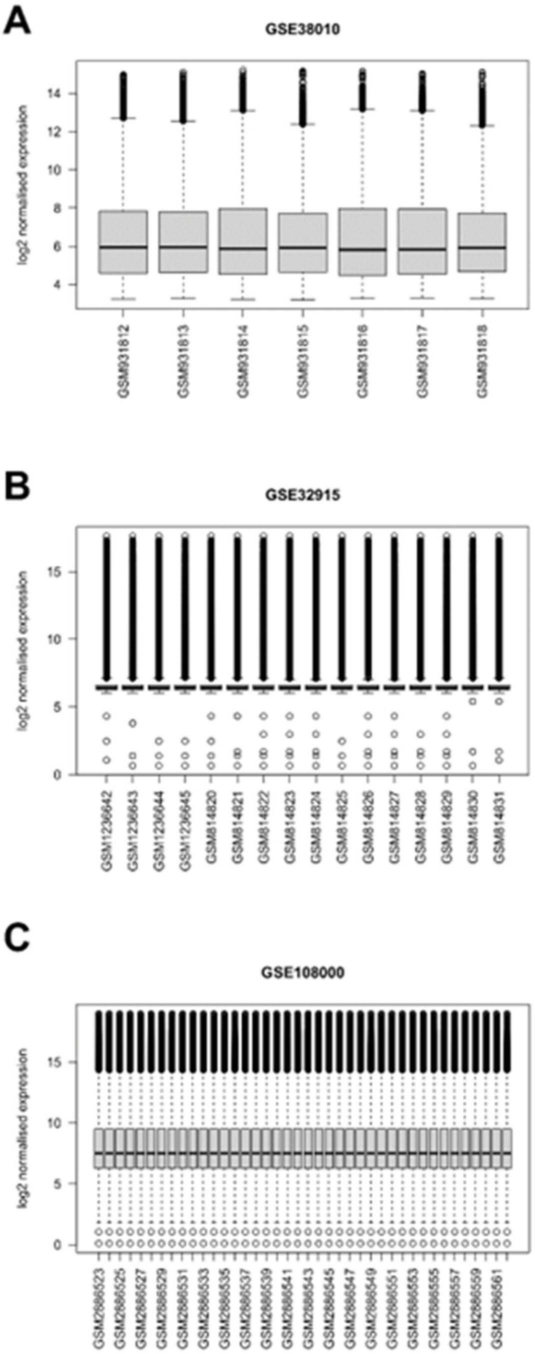

Figure S1. Normalised expression values for each dataset included in the meta-analysis. Boxplots showing normalised and background-corrected expression values for each sample pertaining to (A) GSE38010, (B) GSE32915 and (C) GSE108000 datasets.

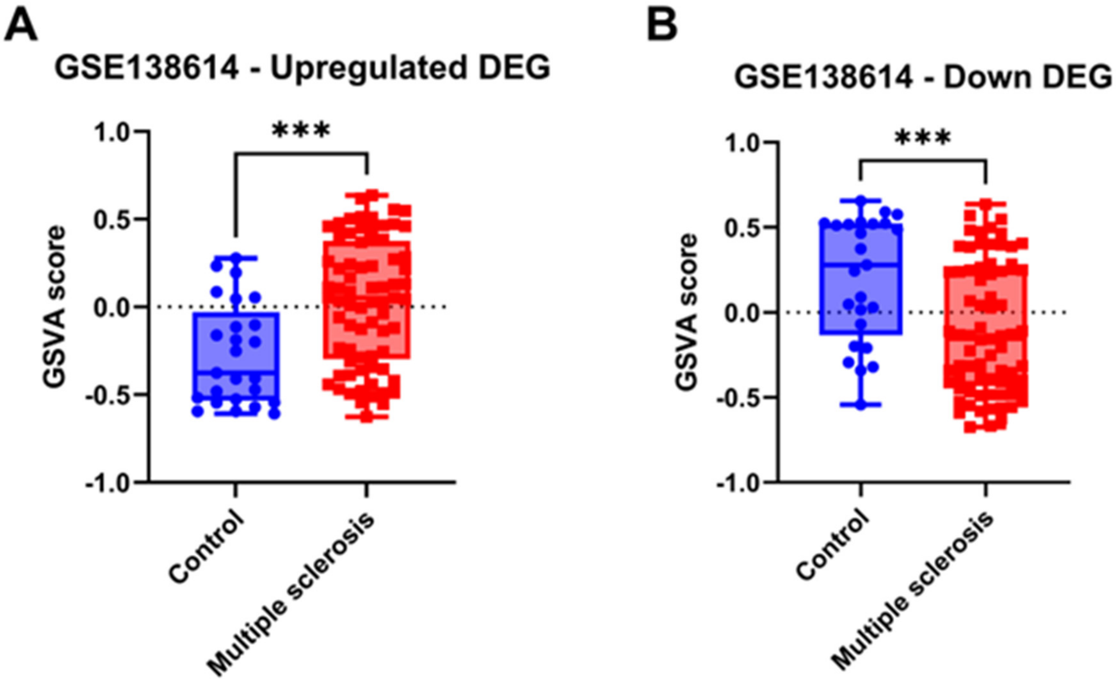

Figure S2. Gene-set variation analysis of the identified DEGs in our meta-analysis. A comparative gene-set variation analysis (GSVA) was performed in the DEGs detected in our meta-analysis and the publicly available RNA-sequencing dataset GSE138614 to assess for similarities in the patterns of gene expression changes. (A) Up-regulated or (B) down-regulated DEGs found in our combined dataset are also significantly upregulated in GSE138614. *** p<0.001 versus Control, as determined by unpaired *t*-test.
